# Initial Response and Outcome of Critically Ill Children With Guillain Barre' Syndrome

**DOI:** 10.3389/fped.2019.00378

**Published:** 2019-09-18

**Authors:** Hafez M. Bazaraa, Hanaa I. Rady, Shereen A. Mohamed, Walaa A. Rabie, Noha H. ElAnwar

**Affiliations:** ^1^Department of Pediatrics, Kasr Alainy Faculty of Medicine, Cairo University, Cairo, Egypt; ^2^Clinical and Chemical Pathology, Kasr Alainy Faculty of Medicine, Cairo University, Cairo, Egypt

**Keywords:** Guillain-Barre' syndrome, inflammatory demyelinating polyneuropathy, axonal neuropathy, flaccid paralysis, critically ill children

## Abstract

**Background:** Guillain-Barre syndrome is the most common cause of acute flaccid paralysis worldwide since the eradication of poliomyelitis. Severe cases may require intensive care and mechanical ventilation.

**Purpose:** was to study pediatric patients with severe GBS requiring intensive care unit (ICU) admission, to assess their course and response to initial treatment modality plasma exchange (PE) or intravenous immunoglobulins (IVIg) and their final outcome.

**Methods:** children with severe GBS who had either actual or impending respiratory failure, bulbar involvement or rapid progression of acute flaccid paralysis with trunk, upper limb and neck involvement within 24 h of the onset of weakness were enrolled.

**Results:** 40 children were included. Following the initial treatment (33 subjects had 5 PE sessions each and IVIg in 7), 16 patients improved (40%), two died and 22 (55%) showed initial treatment failure. Axonal neuropathy, rapid progression and severe motor weakness significantly predicted poor response to therapy. At discharge, favorable outcomes (patient can walk unaided) were present in 22 cases (58%).

**Conclusion:** Despite relatively low mortality, critically ill children with severe GBS have increased prevalence of axonal neuropathy and guarded response to initial therapy with PE or IVIg.

## Introduction

Guillain–Barre' syndrome (GBS) is an acute inflammatory polyneuropathy, characterized by possibly progressive, essentially symmetric, weakness and areflexia; with possible sensory disturbance in a previously well child (1). With an incidence of 4 per 100,000 per year, it is the most common cause of acute flaccid paralysis worldwide since the eradication of poliomyelitis and can affect all age groups (2).

The underlying pathogenesis points to cross reactive immune responses, consequent upon molecular mimicry of certain pathogens with components of the peripheral nerve. In adult population, about a third of patients of GBS require mechanical ventilation (3), up to 20% have severe persistent neurological deficits and approximately 5% die, despite immunotherapy (4).

Cases of pediatric GBS sometimes present with acute-onset rapidly progressive respiratory failure, which can be life-threatening. Disability level in GBS is often measured using the Hughes clinical grading scale (Motor Disability Grading Scale) scored from 1 to 6, with 1 being normal, 5 being the point where patients require mechanical ventilation, and 6 equating with death (5). Early diagnosis and prompt referral for aggressive therapy may significantly improve outcomes (6). Patients with weakness impairing function, any respiratory involvement, or having bulbar insufficiency should start immunotherapy. It had been proved to be efficacious in hastening recovery and improving outcome (7), and should be started as soon as possible, before irreversible nerve damage has taken place (1).

Plasma exchange (PE) and Intravenous immunoglobulins (IVIg) are both effective for treating GBS. PE removes immunoglobulin G autoantibodies and complement, while IVIg can neutralize pathogenic autoantibodies and inhibit consequent complement activation (8). Dramatic improvement within days of starting treatment is not common and if this occurs, it may have happened regardless of treatment. Therefore, a valid approach in milder GBS cases, with no bulbar or respiratory affection, is to closely observe patients for progression in the first 2 weeks while reserving treatment for those who become non-ambulatory or unable to stand unaided (9). The optimum management of the patient with severe GBS who did not improve 10–14 days after PE or IVIg is not yet established.

The aim of our work was to study pediatric patients with severe GBS requiring intensive care unit (ICU) admission, to assess their course and response to initial treatment modality (PE or IVIg) and their final outcome.

## Methods

This is a prospective observational study conducted at the Pediatric Intensive Care Units (PICUs) of Cairo University Pediatric Hospitals over a period of 18 months. During the period between April 2015 and September 2016, all critically ill patients aged between 2 months and 14 years who were admitted with severe GBS were included. All patients had either actual or impending respiratory failure, bulbar involvement or rapid progression of acute flaccid paralysis with trunk, upper limb and neck involvement within 24 h of the onset of weakness. All patients were screened and tested negative for poliovirus infection, per the national poliomyelitis surveillance program.

The study protocol was ethically approved by the research committee of the Department of Pediatrics, Faculty of Medicine, Cairo University and the research ethics committee of Kasr Alainy Faculty of Medicine, Cairo University (approval number I-150114). Informed consents were obtained and documented for all enrolled subjects from their guardians.

### Patients Were Subjected to

#### Initial Assessment

History and clinical examination. Initial laboratory work up included complete blood count, serum electrolytes, CSF examination and immunoglobulin assays when indicated. Hughes scale have been used for semi quantitative assessment of The clinical severity and disability in our patients (5). Motor power was further evaluated on a 0–5 scale for each of upper limbs, lower limbs, trunk and neck as 0 = no movement, 1 = movement with gravity, 2 = movement against gravity, 3 = movement against mild resistance, 4 = movement against moderate resistance, 5 = normal. The total motor power (0–20) was calculated for each patient.

Electro-diagnostic studies were done as soon as feasible after admission after patient stabilization, including electromyography and nerve conduction tests for at least one motor and one sensory nerve in upper and lower limbs. The clinico-pathological type of GBS was classified as either acute inflammatory demyelinating polyneuropathy or acute axonal motor or motor-sensory neuropathy (AIDP, AMAN, or AMSAN, respectively).

#### Initial Treatment

Required supportive care was provided for all patients including cardio-respiratory support, chest care (suctioning and chest physiotherapy), nutritional support, and physical therapy was provided for respiratory and limb muscles after stabilization, to prevent muscle atrophy and contractures.

Five sessions of single-volume PE on alternate days were performed for all eligible patients, using the Gambro Prismaflex machine with isovolumetric substitution with 5% albumin within 24 h of PICU admission.

Intravenous immunoglobulins (IVIg) in a dose of 400 mg/kg for 5 successive days was given to patients weighing <10 kg, those with hemodynamic instability or other contraindications of PE.

#### Assessment of Response and Outcome

The response to initial treatment was assessed 5 days after the last PE session or the last dose of IVIg. Treatment failure was defined as any of the following:

- Failure to extubate a mechanically ventilated patient due to either the need of respiratory support or of endotracheal suctioning and chest care.
- Residual bulbar manifestations.
- Severe residual motor affection of Hughes score 4 or 5.

Patients were followed until discharge from the unit. Further therapies required as well as the final outcome at discharge (survival, length of stay, duration of ventilator support and neurological condition) were recorded.

#### Data Analysis

Data were subjected to statistical analysis using SPSS for windows version 18.0. Nominal data were expressed as frequency and percentage and were compared using Chi square tests. Numerical data were reported as mean and standard deviation (SD) and compared using *t*-tests for parametric data. Non-parametric data were described as median and interquartile range (IQR) and were compared using Mann Whitney *U* tests. Receiver operating characteristics (ROC) curve was used to explore the relation between total motor power on admission and response to initial treatment. *P*-values <0.05 were considered significant.

## Results

Forty cases of severe GBS were included; 21 males and 19 females, with peak presentation was in the months May until August. Thirty required mechanical ventilation, nine presented with bulbar involvement and one with rapidly progressive weakness without bulbar involvement or respiratory failure. Initial treatment consisted of PE sessions in 33 patients and IVIg in seven (17.5%). No adverse effects were noted during PE. Following the initial treatment, 16 patients improved (40%), two died and 22 (55%) showed initial treatment failure (Tables 1, 2).

**Table 1:** Descriptive data of enrolled cases and their initial treatment response.

**Initial therapy**	**PE (*n* = 33)**	**IVIG (*n* = 7)**	***P*-value**
**GBS disability score**				
4 (unable to walk)	6 (18%)	3 (43%)	0.078
5 (needs MV)	27 (82%)	4 (57%)	
**EMG**				
Demyelinating (AIDP)	8 (24%)	2 (29%)	0.41
Axonal (AMAN/ AMSAN)	25 (76%)	5 (71%)	
Age (Mo)^*^	48 (24–87)	18 (14–48)	0.10
Weight (Kg)^*^	15 (11–24.5)	11.3 (6.3–16.5)	0.19
**Initial treatment response**				
Improved	11 (33%)	5 (71%)	0.031^a^
Treatment failure	21 (64%)	1 (14%)	
Early deaths	1 (3%)	1 (14%)	0.11^b^

a *Improved cases vs. those not improved (treatment failure or early death)*.

b *Deaths vs. all survivals after initial treatment*.

Data expressed as frequency (percentage) except

* *as median (IQR)*.

*PE, plasma exchange; IVIG, intravenous immunoglobulins; AIDP, acute inflammatory demyelinating polyneuropathy; AMAN, acute motor axonal neuropathy; AMSAN, acute motor & sensory axonal polyneuropathy; MV, mechanical ventilation*.

**Table 2:** Comparison between clinical condition before vs. after initial treatment.

**Progression**	**Before (*n* = 40)**	**After (*n* = 38)**	***P*-value**
Mechanical ventilation	30 (75%)	14 (37%)	0.0003
Bulbar involvement	37 (93%)	11 (29%)	<0.0001
Extubated + No bulbar	1 (3%)	20 (53%)	<0.0001
Sensory involvement	13 (33%)	3 (8%)	0.004
Autonomic manifestations	13 (33%)	5 (13%)	0.02

**Figure 1:**
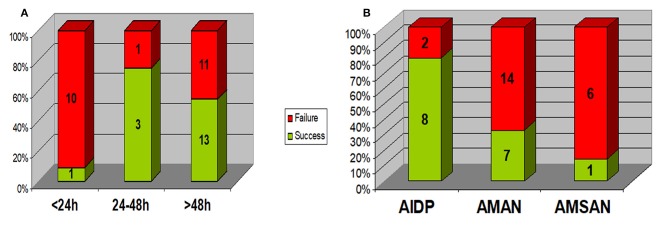
Relation between response to initial treatment and **(A)** disease progression rate (duration of progression) **(B)** EMG pattern. Rapid progression within 24 h was associated with a lower rate of success of initial treatment (1/11 vs. 16/28; *p* = 0.003). Those with AIDP had a higher rate of success of initial treatment (8/10) compared to axonal types (8/28); *p* = 0.02. AIDP, acute inflammatory demyelinating polyneuropathy; AMAN, acute motor axonal neuropathy; AMSAN, acute motor & sensory axonal neuropathy.

As shown in Figure 1, Rapid progression within 24 h was associated with a lower rate of success of initial treatment (1/11 vs. 16/28; *p* = 0.003), 6/11 presented with AMAN, 4/11 presented with AMSAN and 1/11 with AIDP. While those with AIDP had a higher rate of success of initial treatment (8/10) compared to axonal types (8/28); *p* = 0.02.

**Figure 2:**
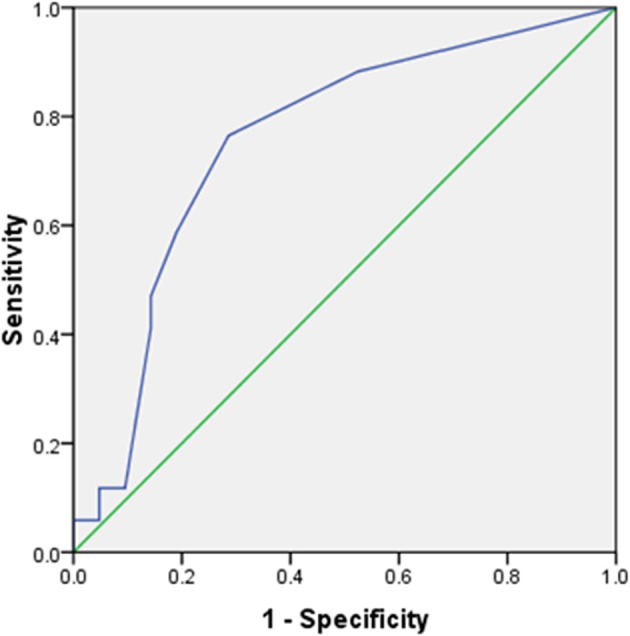
Receiver operating characteristics (ROC) curve for total power at presentation vs. response to initial treatment. AUC 0.755 (95% CI 0.6–0.92); *p* = 0.008. At a cut-off of <2/20, total motor power at presentation predicted initial treatment failure with 76.5% sensitivity, 71.5% specificity.

Total motor power <2/20 at presentation could predict initial treatment failure with a sensitivity of 76.5% and specificity of 71.5% (AUC 0.755 (95% CI 0.6–0.92); *p* = 0.008) (Figure 2). Patients with initial treatment failure required significantly longer ICU stay and MV support (Table 3).

**Table 3:** Duration of support and motor power progression in the study group.

	**Overall**		**Initial treatment failure**		***P*-value**
	**Median**	**IQR**	**Median**	**IQR**	
Admission days	24.5	13–46	43	25–91	<0.001
Mechanical ventilation days	7	0–23	21	9–62	<0.001
Power at presentation	1.5	0–6.25	1	0–2	0.006
Power post-treatment^a^	2.5	0–11.75	2	0–4	0.14
Power at discharge^b^	12	8–14.75	12	6–14	0.26

a P = 0.036 for power improvement before vs. after initial treatment.

b *P = 0.001 for power improvement from immediately following initial treatment to discharge*.

Of those with initial treatment failure, 7/22 received further PE sessions alone and other 15/22 received complementary immunotherapy (steroids ± cyclosporine) with PE sessions. Two deaths occurred early during initial treatment (on day 4 and 6), while three other patients died between days 25–47; representing an overall mortality of 12.5%. Causes of death were sepsis with multiple organ system failure in two cases, pneumonia in two others and Non-Hodgkin lymphoma (the underlying disease) in one case.

**Figure 3:**
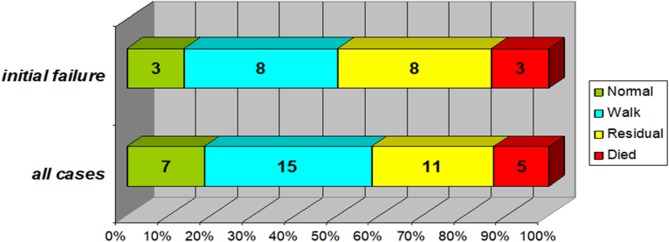
Final outcome of the study group (In the subgroup of patients with initial treatment failure (*n* = 22) compared to the whole study group). Normal: no neurological abnormalities and normal motor power. Walk: can walk unaided (if age-appropriate) despite minor weakness. Residual: significant residual weakness, cannot walk unaided. Favorable outcomes (patient can walk unaided) were present in 22 cases (58%); 7 of them had fully regained their power, while 11 (29%) had been discharged with minor residual weakness and 2 were still admitted. Those with initial treatment failure (top bar) were not significantly different regarding either mortality (*p* = 0.46) or favorable outcome (*p* = 0.12).

At discharge, Favorable outcomes (patient can walk unaided) were present in 22 cases (58%); 7 of them had fully regained their power, while 11 (29%) had been discharged with minor residual weakness and 2 were still admitted. There was no significant difference regarding final outcome between those with initial treatment failure and those without [mortality (*p* = 0.46) or favorable outcome (*p* = 0.12)] (Figure 3).

## Discussion

The most common cause of acute flaccid paralysis worldwide is Guillain Barre Syndrome (2). While some cases can be severe, rapidly progressive and life threatening; prompt supportive treatment with immunotherapy can be effective (10). In this study, 40 patients admitted in the PICUs of Cairo University Pediatric Hospitals with severe GBS were enrolled, 75% of whom required MV.

Consistent with that, the scope of the present study was critically ill, particularly severe, cases; the need for mechanical ventilation in our cases was higher than that was reported in previous studies (11, 12). To the same effect, the vast majority (93%) of studied cases had bulbar involvement. Regarding autonomic manifestations, reported rates reached more than 50% of patients with GBS (13, 14), compared to 33% in our study.

In the current study, patients had severe neurological affection at presentation, further illustrated by the severity of motor weakness. The total power of the upper limbs, lower limbs, trunk and neck had a median of 1.5/20. Moreover, low total power at presentation (<2) was found to significantly predict initial treatment response, with 76.5% sensitivity and 71.5% specificity.

Nevertheless, overall mortality in our study was 13%. Comparable to other reports of children hospitalized with GBS, ranging between 8 and 16% (11, 15). It is; however, notable that four out of five deaths in our series were associated with infectious complications, a possible area for outcome improvement.

Regarding motor neurological recovery, 58% of studied subjects regained normal or essentially normal power with the ability to walk unaided and 29% had significant residual weakness. Complete or almost complete functional recovery was reported in 58% of cases described by Halawa et al. (15), while severe neurological disability in adults was reported in 13% of cases by Rees et al. (16). Better outcomes were reported from Sweden (17). Satisfactory improvement following initial treatment was achieved in 40% of cases. Severe Guillain Barre Syndrome requiring PICU admission should not be considered easily responsive to therapy and outcome is related to disease severity.

In our study we used the Hughes scale for neurological assessment at presentation although we are aware of its limitation to use in infants and small children, however, we couldn't find a similar score specific for this age group, and all our patients were critically ill children with scores 4 and 5 at presentation.

In addition to the severity of motor weakness, rapid progression of weakness within 24 h was associated with higher odds of initial treatment failure. Rapidity of progression of symptoms was associated with poor neurological outcome in other studies as well (18, 19). Among the electrophysiological correlates, axonopathy has been suggested to be predictive of poor neurological outcome and prolonged respiratory paralysis (20) and was associated with rapid progression and worse outcome (21, 22).

Reports from western countries showed that AIDP is the most common subtype of GBS (23, 24), while axonopathy has been the predominant underlying subtype in East Asia and South America (12, 25). The vast majority (75%) of our patients had axonopathy, mostly of the AMAN variant. This could have been confounded by the fact that the current study exclusively enrolled severe cases needing PICU admission. Axonal motor neuropathy is considered a more severe disease variant (22). On the other side, AIDP was shown to have the best response to initial treatment despite they represented a minority of cases admitted to the ICU (25%) during the study period.

Plasmapheresis and IVIg had been described as equally effective therapies for GBS (26); however, several other studies reported a significant decrease in the duration of hospitalization and a significant increase in the number of children with complete recovery in severe cases treated with plasmapheresis (27, 28). This has been explained by that most of patients requiring MV likely have an intense autoantibody production with a high percentage of these antibodies already bound to nerves on development of respiratory failure. This subset of patients would preferentially benefit from removal of antibodies by PE, in comparison to blocking antibody production by IVIg (28). The majority of our patients required MV and initial therapy consisted of PE in most of our cases. Although these did not reach statistical significance, the seven patients who received IVIg tended to be younger, to require less MV and consequently have better initial response. The current study is neither designed nor capable of making conclusions regarding the difference between both therapies.

The use of steroids alone in patients with GBS is highly controversial, with most recent studies showing no beneficial effects, possibly due to the harmful effects of corticosteroids on de-nervated muscle or its inhibition of macrophage repair processes (29). Higher doses of intravenous steroids may be of value and were recommended as add on therapy in treatment of severe or protracted GBS by some authors, possibly in combination with IVIg (30). Treatment of children who fail to respond appropriately to initial therapy remains an important and controversial issue.

In conclusion, children with severe GBS requiring ICU admission show a high proportion of the more severe and resistant axonal type. Despite low mortality, about half of these patients fail to respond adequately to initial specific therapy with PE or IVIg. Rapid progression of weakness within 24 h and low motor power on admission were associated with a poor response. Infections were the main cause of mortality. Finally, favorable neurological outcome on discharge is achieved in 58% of cases. Management of initial treatment failure is a potential area for further study.

## Data Availability

The datasets generated for this study are available on request to the corresponding author.

## Ethics Statement

The study protocol was ethically approved by the research committee of the Department of Pediatrics, Faculty of Medicine, Cairo University and the research ethics committee of Kasr Alainy Faculty of Medicine, Cairo University (approval number I-150114).

## Author Contributions

HB and HR designed the study. SM and NE enrolled patients and collected data. Patient management was coordinated by NE under supervision of HB and HR and laboratory work by WR. Literature review was performed by SM, WR, and NE, and data analysis by HB and WR. All authors approved the study design and the submitted manuscript.

### Conflict of Interest Statement

The authors declare that the research was conducted in the absence of any commercial or financial relationships that could be construed as a potential conflict of interest.

## References

[1] Esposito S Longo MR. Guillain–Barré syndrome. Autoimmun Rev. (2017) 16:96–101. 10.1016/j.autrev.2016.09.022 27666816

[2] Momen AA Shakurnia A. An epidemiological analysis of acute flaccid paralysis in Khuzestan Province, southwest Iran, from 2006 to 2010. Epidemiol Health. (2016) 38:e2016030. 10.4178/epih.e2016030 27457060 PMC5037358

[3] van den Berg B Walgaard C Drenthen J Fokke C Jacobs BC van Doorn PA. Guillain-Barré syndrome: pathogenesis, diagnosis, treatment and prognosis. Nat Rev Neurol. (2014) 10:469–82. 10.1038/nrneurol.2014.121 25023340

[4] Hughes RAC Swan AV Raphaël JC Annane D van Koningsveld R van Doorn PA. Immunotherapy for Guillain-Barré syndrome: a systematic review. Brain. (2007) 130 (Pt 9):2245–57. 10.1093/brain/awm004 17337484

[5] Hughes RAC Newsom-Davis JM Perkin GD Pierce JM Controlled trial of prednisolone in acute polyneuropathy. Lancet. (1978) 312:750–3. 10.1016/S0140-6736(78)92644-2 80682

[6] Medici C Gonzalez G Cerisola A Scavone C. Locked-in syndrome in three children with guillain-barré syndrome. Pediatr Neurol. (2011) 45:125–8. 10.1016/j.pediatrneurol.2011.03.005 21763955

[7] Vitaliti G Tabatabaie O Matin N Ledda C Pavone P Lubrano R. The usefulness of immunotherapy in pediatric neurodegenerative disorders: a systematic review of literature data. Human Vaccines Immunotherapeut. (2015) 11:2749–63. 10.1080/21645515.2015.1061161 26266339 PMC5391617

[8] Sudo M Yamaguchi Y Späth PJ Matsumoto-Morita K Ong BK Shahrizaila N. Different IVIG glycoforms affect *in vitro* inhibition of anti-ganglioside antibody-mediated complement deposition. PLoS ONE. 9:0107772. 10.1371/journal.pone.0107772 25259950 PMC4178036

[9] Dimachkie MM Barohn RJ. Guillain-Barré syndrome and variants. Neurol Clin. (2013) 31:491–510. 10.1016/j.ncl.2013.01.005 23642721 PMC3939842

[10] Chalela JA. Pearls and pitfalls in the intensive care management of Guillain-Barré syndrome. Semin Neurol. (2001) 21:399–405. 10.1055/s-2001-19411 11774055

[11] Koul RL Alfutaisi A. Prospective study of children with Guillain-Barre syndrome. Indian J Pediatr. (2008) 75:787–90. 10.1007/s12098-008-0099-1 18581067

[12] Tang J Dai Y Li M Cheng M Hong S Jiang L. Guillain-Barré syndrome in Chinese children: a retrospective analysis. Pediatr Neurol. (2011) 45:233–7. 10.1016/j.pediatrneurol.2011.06.007 21907884

[13] DiMario FJ Edwards C. Autonomic dysfunction in childhood Guillain-Barré syndrome. J Child Neurol. (2012) 27:581–6. 10.1177/0883073811420872 22241710

[14] Samadi M Kazemi B Golzari Oskoui S Barzegar M. Assessment of autonomic dysfunction in childhood guillain-barre syndrome. J Cardiovasc Thorac Res. (2013) 5:81–5. 10.5681/jcvtr.2013.018 24252981 PMC3825393

[15] Halawa EF Ahmed D Nada MAF. Guillain-Barré syndrome as a prominent cause of childhood acute flaccid paralysis in post polio eradication era in Egypt. Eur J Paediatr Neurol. (2011) 15:241–6. 10.1016/j.ejpn.2010.11.008 21169042

[16] Rees JH Thompson RD Smeeton NC Hughes RA. Epidemiological study of Guillain-Barré syndrome in south east England. J Neurol Neurosurg Psychiatry. (1998) 64:74–7. 10.1136/jnnp.64.1.74 9436731 PMC2169900

[17] Cheng Q Jiang GX Press R Andersson M Ekstedt B Vrethem M. Clinical epidemiology of Guillain-Barré syndrome in adults in Sweden 1996–97: a prospective study. Eur J Neurol. (2000) 7:685–92. 10.1046/j.1468-1331.2000.00128.x 11136356

[18] McKhann GM Griffin JW Cornblath DR Mellits ED Fisher RS Quaskey SA. Plasmapheresis and guillain-barré syndrome: analysis of prognostic factors and the effect of plasmapheresis. Ann Neurol. (2005) 23:347–53. 10.1002/ana.410230406 3382169

[19] Hadden RD Karch H Hartung HP Zielasek J Weissbrich B Schubert J. Preceding infections, immune factors, and outcome in Guillain-Barré syndrome. Neurology. (2001) 56:758–65. 10.1212/WNL.56.6.758 11274311

[20] Sundar U Abraham E Gharat A Yeolekar ME Trivedi T Dwivedi N. Neuromuscular respiratory failure in guillain-barre syndrome: evaluation of clinical and electrodiagnostic predictors. J Assoc Physicians India. (2005) 53:764–8. 16334619

[21] Kumar M Aroor S Mundkur S Kumar S. Guillain-Barré syndrome: a clinical study of twenty children. J Clin Diagnostic Res. (2015) 9:SC09–12. 10.7860/JCDR/2015/8344.5491 25738052 PMC4347143

[22] Wu X Shen D Li T Zhang B Li C Mao M. Distinct clinical characteristics of pediatric guillain-Barré syndrome: a comparative study between children and adults in Northeast China. PLoS ONE. (2016) 11:e0151611. 10.1371/journal.pone.0151611 26974666 PMC4790924

[23] Mitsui Y Kusunoki S Arimura K Kaji R Kanda T Kuwabara S. A multicentre prospective study of Guillain-Barré Syndrome in Japan: a focus on the incidence of subtypes. J Neurol Neurosurg Psychiatry. (2015) 86:110–4. 10.1136/jnnp-2013-306509 24273220

[24] Omejec G Podnar S Retrospective analysis of Slovenian patients with Guillain-Barr?? syndrome. J Peripheral Nervous Syst. (2012) 17:217–9. 10.1111/j.1529-8027.2012.00397.x 22734909

[25] McGrogan A Madle GC Seaman HE de Vries CS. The epidemiology of Guillain-Barré syndrome worldwide. Neuroepidemiology. (2008) 32:150–63. 10.1159/000184748 19088488

[26] Diener HC Haupt WF Kloss TM Rosenow F Philipp T Koeppen S. A preliminary, randomized, multicenter study comparing intravenous immunoglobulin, plasma exchange, and immune adsorption in Guillain-Barré syndrome. Eur Neurol. (2001) 46:107–9. 10.1159/000050777 11528165

[27] Saad K Mohamad IL Abd El-Hamed MA Tawfeek MS Ahmed AE Abdel Baseer KA. A comparison between plasmapheresis and intravenous immunoglobulin in children with Guillain-Barre syndrome in Upper Egypt. Ther Adv Neurol Disord. (2016) 9:3–8. 10.1177/1756285615610471 26788127 PMC4710103

[28] El-Bayoumi MA El-Refaey AM Abdelkader AM El-Assmy MM Alwakeel AA El-Tahan HM. Comparison of intravenous immunoglobulin and plasma exchange in treatment of mechanically ventilated children with Guillain Barré syndrome: a randomized study. Crit Care. (2011) 15:R164. 10.1186/cc10305 21745374 PMC3387601

[29] Verboon C Van Doorn PA Jacobs BC. Treatment dilemmas in Guillain-Barré syndrome. J Neurol Neurosurg Psychiatry. (2017) 88:346–52. 10.1136/jnnp-2016-314862 27837102

[30] van Koningsveld R Schmitz PI Meché FG Visser LH Meulstee J van Doorn PA. Effect of methylprednisolone when added to standard treatment with intravenous immunoglobulin for Guillain-Barré syndrome: randomised trial. Lancet. (2004) 363:192–6. 10.1016/S0140-6736(03)15324-X 14738791

